# Multivariate epidemic count time series model

**DOI:** 10.1371/journal.pone.0287389

**Published:** 2023-06-16

**Authors:** Shinsuke Koyama

**Affiliations:** 1 Department of Statistical Modeling, The Institute of Statistical Mathematics, Tachikawa, Tokyo, Japan; 2 Department of Statistical Science, Graduate University for Advanced Studies (SOKENDAI), Tachikawa, Tokyo, Japan; Stanford University School of Medicine, UNITED STATES

## Abstract

An infectious disease spreads not only over a single population or community but also across multiple and heterogeneous communities. Moreover, its transmissibility varies over time because of various factors such as seasonality and epidemic control, which results in strongly nonstationary behavior. In conventional methods for assessing transmissibility trends or changes, univariate time-varying reproduction numbers are calculated without taking into account transmission across multiple communities. In this paper, we propose a multivariate-count time series model for epidemics. We also propose a statistical method for estimating the transmission of infections across multiple communities and the time-varying reproduction numbers of each community simultaneously from a multivariate time series of case counts. We apply our method to incidence data for the novel coronavirus disease 2019 (COVID-19) pandemic to reveal the spatiotemporal heterogeneity of the epidemic process.

## Introduction

Mathematical and statistical modeling of epidemics is crucial in epidemiology because it provides a theoretical basis for analyzing the spread of infectious diseases and the building blocks of statistical methodologies for data analysis [1, 2]. Epidemic modeling has been used to describe a wide variety of phenomena. For instance, the spread of information, opinions, and social behaviors can be modeled as a contagion process [3]. Epidemic modeling lies at the core of a research field that crosses different disciplines. In this study, we developed a multivariate time-series epidemic analysis model.

During an ongoing pandemic, the most commonly available type of data is the daily (or weekly) number of newly reported cases. The time series of case counts provides information on the epidemic size and transmissibility trends. The time-varying effective reproduction number was defined as the expected number of secondary cases arising from a single primary case. They are widely used to monitor these trends. Recent developments in epidemiological data analysis have utilized statistical methodologies to improve the efficiency of estimating the effective reproduction number [4, 5]. However, most existing methods are limited to univariate time-series analyses. Although epidemics spread across multiple communities, populations, or regions, these methods can only be used to calculate the univariate effective reproduction numbers of each community separately. To incorporate infection transmission over multiple communities, it is necessary to extend these methods to perform multivariate analysis.

In this study, we propose a multivariate count time series model that describes an epidemic process in multiple nodes. Central to our approach is introducing latent variables to represent successive infections in transmission chains. These latent variables enable us to make posterior inferences regarding the transmission of infections across multiple nodes and develop an EM-type algorithm for estimating the model parameters. In particular, the proposed algorithm can be used to simultaneously estimate the entries in the adjacency matrix and time-varying effective reproduction numbers of each node from a multivariate incidence time series. An application of our method is demonstrated using synthetic and actual data from the COVID-19 pandemic.

### Related works

The effective reproduction number can be defined in two approaches: instantaneous and cohort reproduction numbers. The former measures transmission at a specific point in time, while the latter measures transmission in a specific cohort of individuals [6, 7]. In the following methods, either of these reproduction numbers are estimated from a time series of case counts.

Wallinga and Teunis’s method is based on the likelihood of a renewal model and is commonly used to estimate the cohort reproduction number [8]. In the methods proposed by [9] (EpiEstim) and Bettencourt and Ribeiro [10], the instantaneous reproduction number is estimated using a Bayesian framework. The main difference between these two methods lies in the model assumption. A renewal model is assumed in EpiEstim, similar to the Wallinga and Teunis method, whereas the Bettencourt and Ribeiro method is based on the linearized growth rate of a SIR model. Compared to the SIR model, the renewal model involves simpler parametric assumptions regarding the epidemic process and requires only the generation interval distribution. An advantage of methods based on the renewal model is their simplicity: Parsimony reduces the risk of model misspecification when there are many unknowns in the underlying process. A comprehensive comparison of these three methods was presented by [7].

In a recently proposed method [4], the cohort reproduction number was estimated and combined with the state-space model in a renewal model, and the estimation problem was solved using a recursive Bayesian smoothing procedure. Parag [5] independently proposed a method to estimate the instantaneous reproduction number along the same lines. To the best of our knowledge, these are state-of-the-art methods for estimating the effective reproduction number from an incidence curve.

However, note that all these methods apply only to univariate time-series data.

## Methods

### Multivariate-count time series model

First, we consider a univariate epidemic model. Let *n*_*t*_ be the number of cases in which symptoms begin at time *t*. Given an initial case at time *t* = 1, the rate of new cases at time *t*(≥ 2) can be described as

$$\begin{array}{c} \lambda_{t}=\sum_{\tau =1}^{t-1}R_{t-\tau }\phi_{\tau }n_{t-\tau }, \end{array}$$ (1)

where {*ϕ*_*τ*_} is the serial interval distribution (i.e., the distribution of time from symptom onset in the primary case to symptom onset in the secondary case [11]) and *R*_*t*−*τ*_ is the effective reproduction number at time *t* − *τ*.

Before extending it to perform multivariate analysis, we contrast Eq (1) with the conventional renewal model that describes epidemic processes [5, 6, 9, 12].

$$\begin{array}{c} \lambda_{t}=R_{t}\sum_{\tau =1}^{t-1}\phi_{\tau }n_{t-\tau }. \end{array}$$ (2)

The most significant difference between these two models lies in their treatment of the effective reproduction number. Here, *R*_*t*_ in Eq (2) represents the instantaneous reproduction number, while *R*_*t*−*τ*_ in Eq (1) represents the cohort reproduction number, as shown in S1 File. Additionally, the count *n*_*t*−*τ*_ in Eq (2) represents the number of cases infected at time *t* − *τ*; consequently, {*ϕ*_*τ*_} represents the generation time distribution. Therefore, to apply this model to reported cases, it is necessary to adjust the time lag between infection and symptom onset [6]. By contrast, because the case definition in Eq (1) is based on symptoms, there is no need to adjust for the time lag. Hence, we employ Eq (1) as the basis of our multivariate model.

We consider an infection process that spreads over *D* nodes, where each node represents a group of individuals. Let *n*_*it*_ be the number of newly reported cases in (*i*, *t*), where (*i*, *t*) denotes node *i* at time *t*. By extending Eq (1) to a multivariate setting, the rate of new cases at (*i*, *t*) is given by

$$\begin{array}{c} \lambda_{it}=\sum_{j=1}^{D}a_{ij}\sum_{\tau =1}^{t-1}R_{j,t-\tau }\phi_{\tau }n_{j,t-\tau }, \end{array}$$ (3)

where *R*_*j*,*t*−*τ*_ denotes the effective reproduction number at (*j*, *t* − *τ*) and *a*_*ij*_(≥ 0) represents the transmission ratio from node *j* to node *i* satisfying $\sum_{i=1}^{D}a_{ij}=1$. Based on the history of previously reported cases,

$$\begin{array}{c} N_{1:t-1}\equiv \left\{n_{js}|j=1,\ldots ,D;s=1,\ldots ,t-1\right\}, \end{array}$$ (4)

the count *n*_*it*_ is assumed to follow a Poisson distribution with the rate Eq (3):

$$\begin{array}{c} P\left(n_{it}|N_{1:t-1}\right)=\text{Poisson}\left(\lambda_{it}\right)\equiv \frac{\lambda_{it}^{n_{it}}}{n_{it}!}e^{-\lambda_{it}}. \end{array}$$ (5)

The multivariate count time series model comprises the two components in Eqs (3) and (5).

### Latent variable representing secondary infection in transmission chain

Now, let us introduce the latent variable $y_{it}^{js}$, which represents the number of secondary cases at (*i*, *t*) infected by the primary cases at (*j*, *s*) (*s* < *t*). As the total number of new cases at (*i*, *t*) is given by *n*_*it*_, the following equality holds:

$$\begin{array}{c} \sum_{j=1}^{D}\sum_{s=1}^{t-1}y_{it}^{js}=n_{it}. \end{array}$$ (6)

Assuming conditional independence between the transmission events $y_{it}^{js}$ and $y_{it}^{j^{\prime }s^{\prime }}$ for (*j*, *s*) ≠ (*j*′, *s*′) and given *N*_1:*t*−1_. The superposition principle can be applied to the Poisson distribution (5), leading to a Poisson distribution for counts $y_{it}^{js}$ with the rate given by $\psi_{it}^{js}=a_{ij}R_{js}\phi_{t-s}n_{js}$. Thus, given the sum of independent Poisson random variables *n*_*it*_, the conditional distribution of each element of the Poisson vector $Y_{it}\equiv \left\{y_{it}^{js}|j=1,\ldots ,D;s=1,\ldots ,t-1\right\}$ is multinomially distributed with count probabilities scaled by the sum of the individual rates:

$$\begin{array}{c} P\left(Y_{it}|n_{it},N_{1:t-1}\right)=\frac{n_{it}!}{\prod_{j=1}^{D}\prod_{s=1}^{t-1}y_{it}^{js}!}\prod_{j=1}^{D}\prod_{s=1}^{t-1}(\frac{\psi_{it}^{js}}{\lambda_{it}}{)}^{y_{it}^{js}}. \end{array}$$ (7)

In particular, the conditional expectation of $y_{it}^{js}$, given *n*_*it*_ and *N*_1:*t*−1_, is

$$\begin{array}{c} \left\langle y_{it}^{js}\right\rangle \equiv E\left(y_{it}^{js}|n_{it},N_{1:t-1}\right)=\frac{n_{it}\psi_{it}^{js}}{\lambda_{it}}=\frac{n_{it}a_{ij}R_{js}\phi_{t-s}n_{js}}{\sum_{k=1}^{D}a_{ik}\sum_{s=1}^{t-1}R_{ks}\phi_{t-s}n_{ks}}, \end{array}$$ (8)

from which posterior inferences can be made regarding secondary infections from the reported incidences.

### Parameter estimation

Using latent variables, we develop an expectation maximization (EM)-type algorithm to estimate the model parameters. In this study, we focus on estimating the weighted adjacency matrix *A* = (*a*_*ij*_) and time-varying reproduction number *R* = {*R*_*js*_} of each node from an observed multivariate time series of incidence *N*_1:*T*_. To estimate these parameters, we consider the following penalized log-likelihood function:

$$\begin{array}{ccc} L(A,R) & = & \sum_{i=1}^{D}\sum_{t=1}^{T}\;\text{log}\;P\left(n_{it}|N_{1:t-1}\right)-\gamma \sum_{j=1}^{D}\sum_{s=2}^{T}{\left|R_{js}-R_{j,s-1}\right|}^{p}, \end{array}$$ (9)

where the choice of exponent *p* ∈ {1, 2} and hyperparameter *γ* ≥ 0 depend on the sparsity or smoothness of the variation in the time-varying reproduction numbers.

Rather than maximizing Eq (9) with respect to *A* and *R* directly, we iteratively update the estimates to ensure that the objective function L(A,R) increases monotonically. To update the parameters, we construct a tight lower bound *Q*(*A*, *R*|*A*^(*k*)^, *R*^(*k*)^) for the current parameter estimations {*A*^(*k*)^, *R*^(*k*)^} such that

$$\begin{array}{ccc} Q(A,R|A^{\left(k\right)},R^{\left(k\right)}) & \leq & L(A,R)\;\text{for}\;\text{all}\;A\;\text{and}\;R, \end{array}$$ (10)

$$\begin{array}{ccc} Q(A^{\left(k\right)},R^{\left(k\right)}|A^{\left(k\right)},R^{\left(k\right)}) & = & L(A^{\left(k\right)},R^{\left(k\right)}). \end{array}$$ (11)

Maximizing the function that satisfies these properties ensures that the objective function increases monotonically. Similar to the EM algorithm, the lower bound is obtained as follows:

$$\begin{array}{ccc} Q(A,R|A^{\left(k\right)},R^{\left(k\right)}) & = & \sum_{i=1}^{D}\sum_{t=1}^{T}\sum_{j=1}^{D}\sum_{s=1}^{t-1}\{{\left\langle y_{it}^{js}\right\rangle }^{\left(k\right)}\;\text{log}\left(a_{ij}R_{js}\phi_{t-s}n_{js}\right)\\ & & -a_{ij}R_{js}\phi_{t-s}n_{js}\}-\gamma \sum_{j=1}^{M}\sum_{s=2}^{T}{\left|R_{js}-R_{j,s-1}\right|}^{p}, \end{array}$$ (12)

where ${\langle y_{it}^{js}\rangle }^{\left(k\right)}$ is the conditional expectation of $y_{it}^{js}$ computed from Eq (8) and the current parameter estimations {*A*^(*k*)^, *R*^(*k*)^}. (See S1 File for the derivation.)

**Updating of** *A* The update for *a*_*ij*_ is obtained by maximizing Eq (12) with respect to *a*_*ij*_ under constraint $\sum_{i=1}^{D}a_{ij}=1$. Using the Lagrange multiplier method, we obtain

$$\begin{array}{c} a_{ij}^{\left(k+1\right)}=\frac{\sum_{t=1}^{T}\sum_{s=1}^{t-1}{\left\langle y_{it}^{js}\right\rangle }^{\left(k\right)}}{\sum_{i=1}^{D}\sum_{t=1}^{T}\sum_{s=1}^{t-1}{\left\langle y_{it}^{js}\right\rangle }^{\left(k\right)}}. \end{array}$$ (13)

Eq (13) leads to a natural interpretation of *a*_*ij*_ as the fraction of the expected number of secondary cases in node *i* infected by the primary cases in node *j*.

**Updating of** *R* The update of *R*_*js*_ satisfies the following equation:

$$\begin{array}{c} \frac{\partial }{\partial R_{js}}Q\left(A,R|A^{\left(k\right)},R^{\left(k\right)}\right)\;\\ =\frac{{\left\langle y^{js}\right\rangle }^{\left(k\right)}-\nu_{s}R_{js}n_{js}}{R_{js}}-\gamma \frac{\partial }{R_{js}}\{|R_{j,s+1}-R_{js}{|}^{p}+{\left|R_{js}-R_{j,s-1}\right|}^{p}\}=0, \end{array}$$ (14)

where ${\langle y^{js}\rangle }^{\left(k\right)}\equiv \sum_{i=1}^{D}\sum_{t=s+1}^{T}{\langle y_{it}^{js}\rangle }^{\left(k\right)}$ denotes the total number of expected secondary infections caused by primary cases at (*j*, *s*), and $\nu_{s}=\sum_{\tau =1}^{T-s}\phi_{\tau }$. As shown in S1 File, the solution of the system of the above equations (*s* = 1, …, *T*) corresponds to the maximum a posteriori (MAP) estimates of a univariate state-space model with a Poisson observation model,

$$\begin{array}{c} {\left\langle y^{js}\right\rangle }^{\left(k\right)}|R_{js}∼\text{Poisson}\left(\nu_{s}R_{js}n_{js}\right), \end{array}$$ (15)

and the state-transition density,

$$\begin{array}{c} p\left(R_{js}|R_{j,s-1}\right)\propto \text{exp}\{-\gamma |R_{js}-R_{j,s-1}{|}^{p}\}. \end{array}$$ (16)

The transition density corresponding to the penalty function is given by a Laplace (*p* = 1) or Gaussian distribution (*p* = 2). The update of the time-varying reproduction number of node *j*, ${\left\{R_{js}^{\left(k+1\right)}\right\}}_{s=1}^{T}$, is then computed using state smoothing for the equivalent state-space model. The details of the smoothing algorithm are provided in S1 File.

The overall algorithm is summarized in Algorithm 1.

**Algorithm 1** Algorithm for estimating *A* and *R*

**Input**: Time series of case counts *N*_1:*T*_, penalty function *p* = 1 or 2, and hyper-parameter *γ* > 0.

1: Initialize $a_{ij}^{\left(0\right)}=1/D$ for all *i*, *j*, and $R_{js}^{\left(0\right)}=1$ for all *j*, *s*.

2: **while** *k* = 0, 1, … **do**

3:  Compute ${\langle y_{it}^{js}\rangle }^{\left(k\right)}$ using Eq (8) with *A*^(*k*)^ and *R*^(*k*)^ for all *i*, *t*, *j*, *s*.

4:  Update $a_{ij}^{\left(k+1\right)}$ using Eq (13) for all *i*, *j*.

5:  **for** *j* = 1 to *D* **do**

6:   Update ${\left\{R_{js}^{\left(k+1\right)}\right\}}_{s=1}^{T}$ by state smoothing.

7:  **end for**

8: **end while**

**output**: *A*^(*k*+1)^, *R*^(*k*+1)^.

The free energy (negative log-marginal likelihood) of the state-space model can be computed through state smoothing (see S1 File for details). We use the total free energy, that is, the sum of the free energies of all the nodes, as the criterion for selecting the penalty function (*p* = 1 or 2) and the value of the hyperparameter *γ*.

## Results

### Analysis of synthetic data

**Fig 1:**
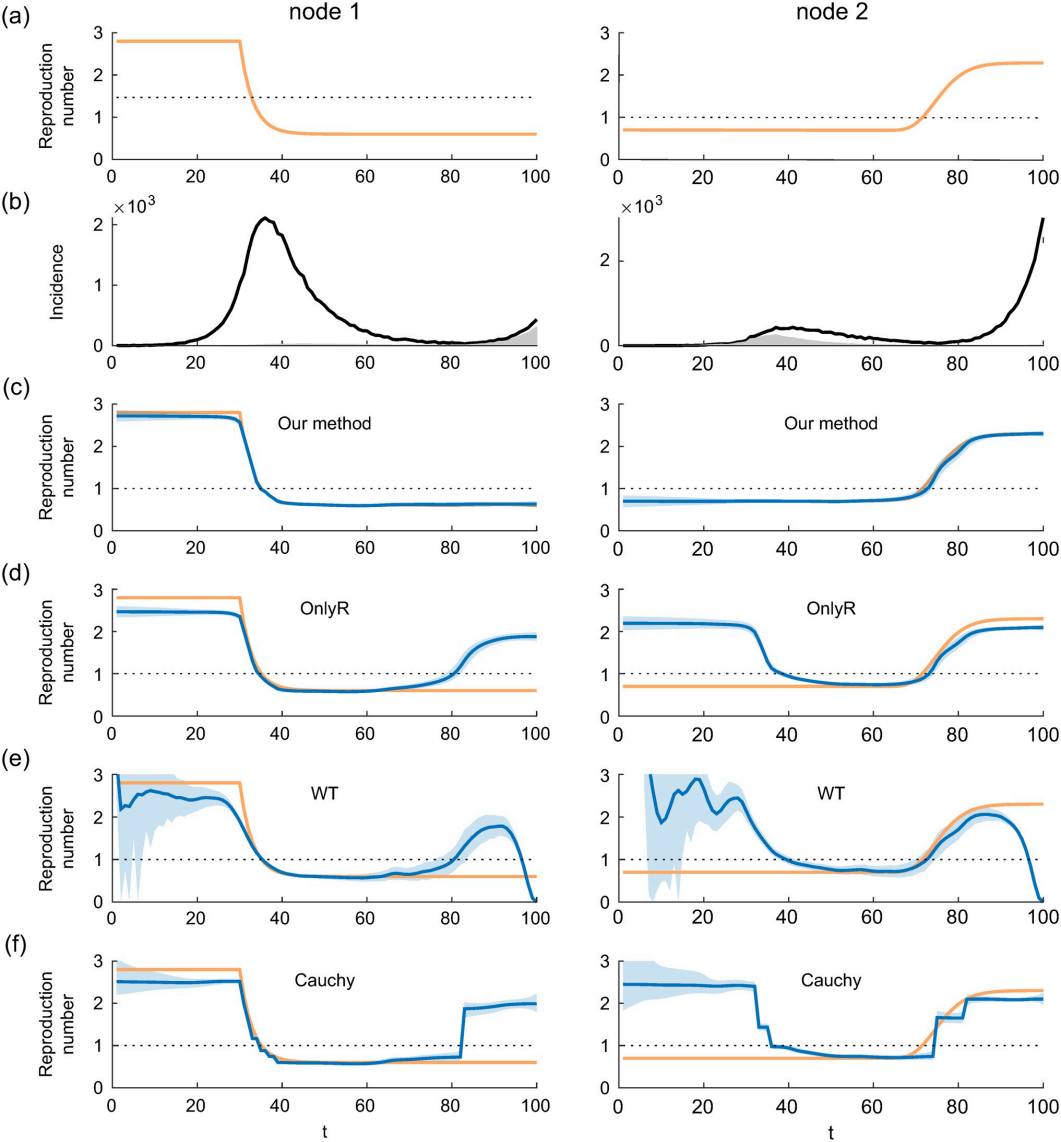
Simulation result. (a) Time-varying reproduction numbers used in the simulation. (b) Simulated infection counts (black line) and estimated infection counts transmitted from the other node (gray area). (c-f) True (yellow line) and estimated reproduction numbers (blue line) with 95% credible interval (shaded area) obtained using our method (c), OnlyR (d), WT (e), and Cauchy (f). Our method successfully estimated the amplitude of the effective reproduction number while the other three methods failed.

We first demonstrate our method using a toy example whereby the infection process is simulated using two nodes (*D* = 2). To mimic the COVID-19 pandemic, we employed a log-normal distribution for the serial interval distribution {*ϕ*_*t*_} with a mean and standard deviation of 4.7 and 2.9 days, respectively, [13]. The transmission ratios were set as *a*_11_ = *a*_22_ = 0.9 and *a*_12_ = *a*_21_ = 0.1. The time-varying reproduction numbers of each node are shown in Fig 1a. Using these parameters and a given initial infection at *t* = 1, the model given by Eqs (3) and (5) is used to generate *N*_1:*T*_. In Fig 1b, we plot the simulated case counts *N*_1:*T*_ for each node (black lines). Because of the profile of the effective reproduction numbers, the incidence curves for both nodes exhibit two waves: the first and second are caused by nodes 1 and 2, respectively.

From the simulated case counts *N*_1:*T*_, we set the penalty function (*p* = 1 or 2) and the value of the hyperparameter *γ* based on free energy minimization using a grid search and estimated the model parameters $\left\{\hat{A},\hat{R}\right\}$. The estimated transmission ratios are ${\hat{a}}_{11}=0.8930$, ${\hat{a}}_{22}=0.9047$, ${\hat{a}}_{12}=0.0953$ and ${\hat{a}}_{21}=0.1070$, which are in good agreement with the true values. Fig 1c shows the estimated time-varying reproduction numbers (blue line) of each node with 95% credible intervals (shaded area), along with the true values (yellow line), from which we confirmed that the amplitudes of the reproduction numbers were properly estimated.

Using the estimated parameters and Eq (8), the conditional expectation of the secondary infections, $\left\{\langle y_{it}^{js}\rangle \right\}$, was computed, from which we estimated the number of infections that are transmitted from the other node, $\langle y_{it}\rangle \equiv \sum_{j\neq i}\sum_{s=1}^{t-1}\langle y_{it}^{js}\rangle$ (Fig 1b, gray area). As expected, we observed that the second wave in node 1 was dominated by infections transmitted from node 2, whereas the first wave in node 2 was initiated by the outbreak of node 1.

To examine the effect of overlooking inter-node infections on estimates of time-varying reproduction numbers, we estimated *R* with the identity matrix for *A* fixed, that is, separate estimates for each node (Fig 1d; ‘OnlyR’). The estimated reproductive numbers are substantially biased. The reproduction number of node 1 during the first wave was underestimated because ignoring the infections node 1 transmitted to node 2, whereas the reproduction number during the second wave was overestimated because it counted the infections transmitted from node 2. (The same holds for the estimated reproduction number of node 2.)

For comparison, we applied two other estimation methods to estimate the time-varying reproduction numbers. The first method is that proposed by Wallinga and Teunis (‘WT’), which is widely used to estimate effective reproduction numbers [8]. In the second method [4], the effective reproduction number is estimated using the state-space method equipped with a Cauchy transition density (“Cauchy”). As these methods are applicable only to univariate time series, the time-varying reproduction numbers for each node are estimated separately. The estimation results obtained using these two methods are plotted in Fig 1e and 1f. As was the case with OnlyR, upward and downward biases were observed in these two methods because the inter-node infections were ignored. The difference in the estimated reproduction numbers among these three methods was attributed to the smoothness assumption made in these estimation methods. In particular, the WT is easily influenced by data fluctuations in the initial phase of the epidemic, and the estimated reproduction numbers decrease at the end of the recorded interval owing to right truncation.

We performed an additional numerical study, in which the number of nodes (dimensions) was varied from *D* = 10 to 50. The transmission ratios were randomly chosen and normalized such that $\sum_{i=1}^{D}a_{ij}=1$, and the time-varying reproduction numbers of each node were randomly chosen between the two profiles, as shown in Fig 1a. To quantify the estimation performance, we compute the average relative error as follows:

$$\begin{array}{c} \frac{1}{DT}\sum_{j=1}^{D}\sum_{s=1}^{T}|R_{js}-{\hat{R}}_{js}\left|/\right|R_{js}|, \end{array}$$ (17)

for time-varying reproduction numbers. We performed the numerical study 10 times with different samples and reported the average performance metrics over the 10 runs. The results are summarized in Table 1. Our method outperformed the other three methods over the range of dimensions examined. This confirmed that the estimation of time-varying reproductive numbers can be significantly improved by considering the transmission of infections across multiple nodes.

**Table 1:** Average relative error between the true *R* and its estimate $\hat{R}$ obtained using our method, OnlyR, WT, and Cauchy.

*D*	Our method	OnlyR	WT	Cauchy
10	0.22 ± 0.02	0.66 ± 0.03	0.74 ± 0.04	0.80 ± 0.02
20	0.28 ± 0.03	0.63 ± 0.02	0.74 ± 0.03	0.81 ± 0.02
30	0.28 ± 0.02	0.57 ± 0.03	0.79 ± 0.13	0.82 ± 0.03
40	0.27 ± 0.02	0.71 ± 0.02	0.77 ± 0.07	0.79 ± 0.01
50	0.28 ± 0.04	0.70 ± 0.03	0.78 ± 0.09	0.81 ± 0.01

### Analysis of actual data

We applied our method to actual data from the COVID-19 pandemic in Japan (https://www.mhlw.go.jp/stf/covid-19/open-data.html). The data consist of newly confirmed cases in *D* = 47 prefectures between January 16, 2020, and November 24, 2021, in which 1,720,441 cases were reported. The data for each week were aggregated to reduce the influence of daily noise on the reported cases. We applied our estimation method to new weekly cases to estimate the parameters $\left\{\hat{A},\hat{R}\right\}$. We empirically confirmed that the algorithms with different initializations converged to the same estimate. Using the estimated parameters and Eq (8), the conditional expectation of secondary infections, $\left\{\langle y_{it}^{js}\rangle \right\}$, was computed, from which we made posterior inferences regarding the infections transmitted across the prefectures. In particular, we computed the expected total number of secondary infections in prefecture *i* that were transmitted from prefecture *j*: $\langle y_{i}^{j}\rangle \equiv \sum_{t=1}^{T}\sum_{s=1}^{t-1}\langle y_{it}^{js}\rangle$.

**Fig 2:**
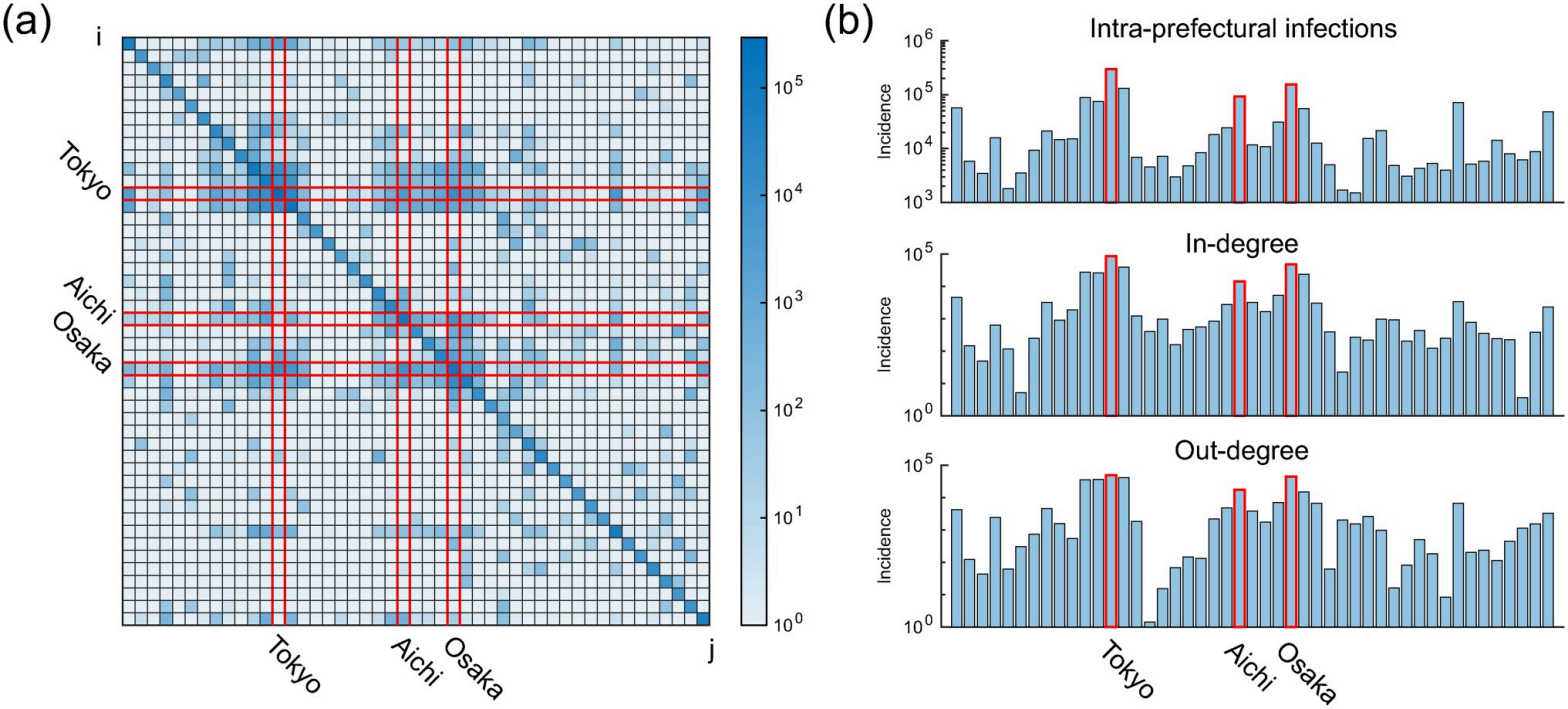
Result of the actual data analysis. (a) Matrix visualization of estimated secondary infections $\left(\langle y_{i}^{j}\rangle \right)$ plotted in log scale. Diagonal and off-diagonal elements represent intra-prefectural and inter-prefectural infections, respectively. (b) Bar graph of intra-prefectural infections $\langle y_{i}^{i}\rangle$ (top), in-degree 〈*y*_*i*_〉 (middle), and out-degree 〈*y*^*j*^〉 (bottom) of each prefecture.

Overall, it was estimated that 82% of the infected cases ($\sum_{i}\langle y_{i}^{i}\rangle =1,414,104$ cases) were infected within each prefecture (“intra-prefectural infections”) and that 18% ($\sum_{i}\sum_{j\neq i}\langle y_{i}^{j}\rangle =306,337$ cases) of the infections were transmitted across prefectures (“inter-prefectural infections”). Fig 2 shows a matrix visualization of $\left(\langle y_{i}^{j}\rangle \right)$ (Fig 2a, heat map) and bar graphs of the intra-prefectural infections $\langle y_{i}^{i}\rangle$, in-degree $\langle y_{i}\rangle \equiv \sum_{j\neq i}\langle y_{i}^{j}\rangle$, and out-degree $\langle y^{j}\rangle \equiv \sum_{i\neq j}\langle y_{i}^{j}\rangle$ in each prefecture (Fig 2b). The prefectures are arranged in geographical order from northeast to southwest. Inter-prefectural infections exhibit a community structure that is correlated with the demographic, economic, and industrial characteristics of the prefectures. The largest community was centered around Tokyo (the capital of Japan), the second-largest community around Osaka (the largest prefecture in the west), and the third-largest community was centered around Aichi (the largest industrial area).

**Fig 3:**
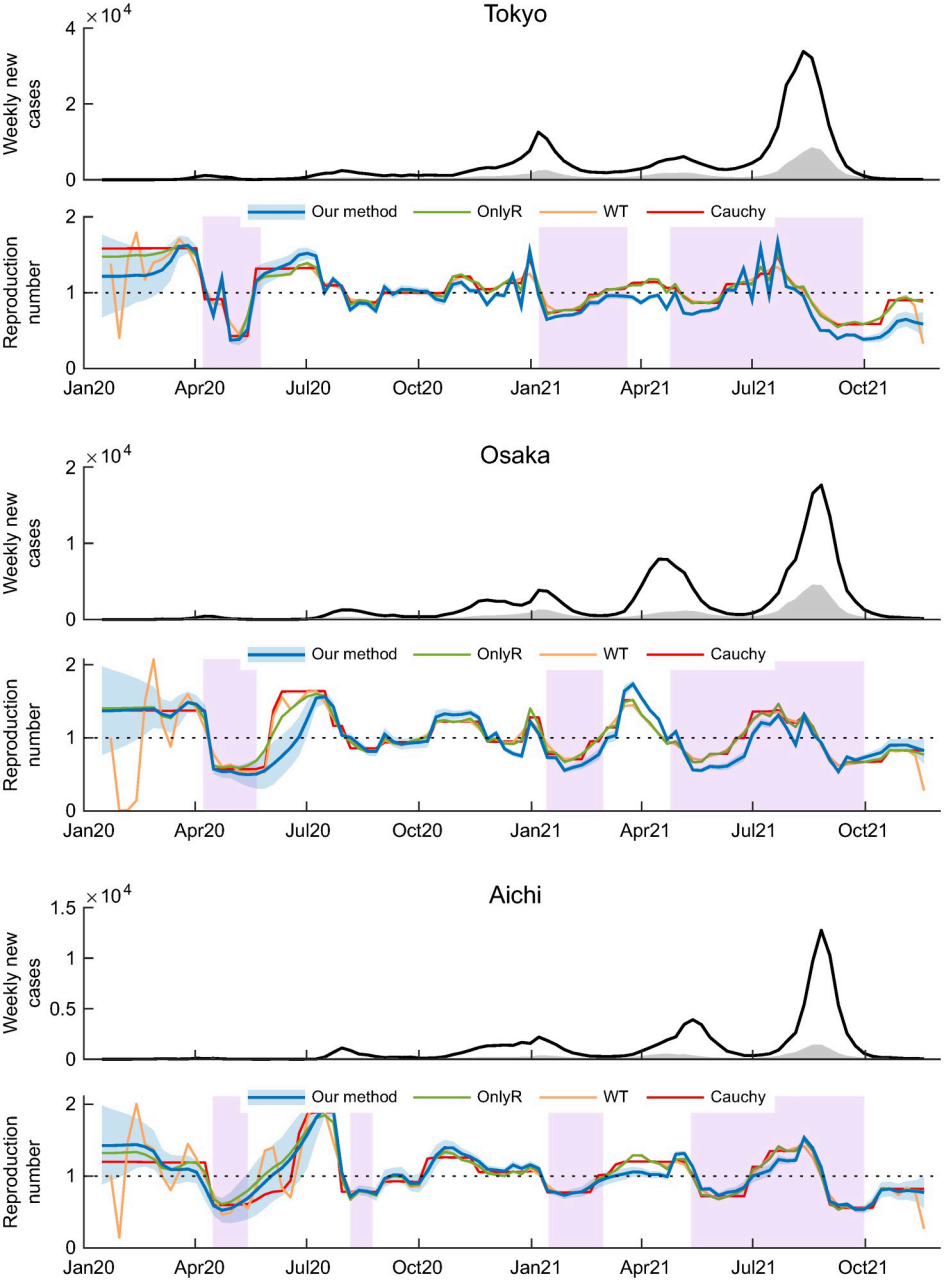
Result of the actual data analysis. Weekly incidence curve (black line) along with estimated infection counts transmitted from outside (gray area) and estimated time-varying reproduction number (blue line) with 95% credible interval (shaded sky blue area) for Tokyo (top), Osaka (middle) and Aichi (bottom) prefectures. Green, yellow, and red lines represent time-varying reproduction numbers estimated by OnlyR, WT, and Cauchy, respectively. The purple range represents the period whereby a state of emergency was implemented.

Fig 3 shows the weekly incidence curves (black lines) along with the estimated infection counts transmitted from the other nodes (gray areas) and estimated time-varying reproduction numbers (blue line) for Tokyo, Osaka, and Aichi. The estimated reproduction numbers exhibited rises and falls that are correlated with the periods whereby the state of emergency was implemented (purple range). In the same figure, we plot the time-varying reproduction numbers estimated using OnlyR (green line), WT (yellow line), and Cauchy (red line). These three estimates exhibit a systematic deviation from that obtained by our method, which indicates that there is a substantial number of inter-prefectural infections transmitted across prefectures. The estimated time-varying reproduction numbers for all the 47 prefectures are shown in S1 Fig.

## Conclusion

In this paper, we proposed a multivariate-count time-series model for epidemics spreading across multiple nodes. The central concept of our approach is to introduce latent variables that represent secondary infections in transmission chains. This enabled us to infer infection transmission across multiple nodes and develop an EM-type algorithm for estimating model parameters. The proposed algorithm simultaneously estimates the weighted adjacency matrix and time-varying reproduction numbers for each node from a multivariate time series of incidence. In addition, we formulated a state-smoothing algorithm to estimate time-varying reproduction numbers. This enabled us to use a tool developed for state-space models.

Because the serial interval distribution is fixed, our estimation method is limited to incidence data whereby the statistical properties of the serial interval remain unchanged. While we employed the log-normal distribution estimated in the early phase of COVID-19 [13], the omicron variant, which became prevalent in January 2022, spreads more easily than the earlier variants of SARS-CoV-2 [14]. Therefore, it is necessary to revise the estimate of the serial interval distribution to assess the transmissibility during distinct phases of the pandemic.

Although we assume that the entire outbreak is driven by transmission within the network, the proposed model (3) can include case imports from outside the network as

$$\begin{array}{c} \lambda_{it}=\mu_{it}+\sum_{j=1}^{D}a_{ij}\sum_{\tau =1}^{t-1}R_{j,t-\tau }\phi_{\tau }n_{j,t-\tau }, \end{array}$$ (18)

where *μ*_*it*_ is the import rate in case (*i*, *t*). Accordingly, the cases imported from outside the network, denoted by $y_{it}^{\prime }$, are incorporated into Eq (6) as

$$\begin{array}{c} y_{it}^{\prime }+\sum_{j=1}^{D}\sum_{s=1}^{t-1}y_{it}^{js}=n_{it}, \end{array}$$ (19)

for which posterior inference based on the multinomial distribution (7) is applied to differentiate between cases arising from the network and those imported from outside. The rate of case importations, *μ*_*it*_, may be pre-estimated by medical inspection during airport quarantine; if this is not the case, simultaneous estimation of *μ*_*it*_, along with the adjacency matrix and time-varying reproduction numbers, will be developed, which is left for future work.

The concept of introducing latent variables to represent transmission chains is similar to that of the branching representation of the Hawkes process [15–17]. A Hawkes-type point process was obtained in the continuous-time limit of the count time-series model. From this perspective, our model can be regarded as its discrete-time counterpart and provides a basis for its application in the analysis of multivariate count time series.

## Supporting information

S1 File Supplementary material to the manuscript. (PDF)

S1 Fig Estimated effective reproduction numbers for 47 prefectures. (PDF)
