# Supplementary material for: Multivariate epidemic count time series model
Source: PLoS One. 2023 Jun 16;18(6):e0287389. doi: 10.1371/journal.pone.0287389 (PMC10275427; doi:10.1371/journal.pone.0287389)
Supplement: S1 File — (PDF) [file pone.0287389.s001.pdf]

Supporting Information to : Multivariate Epidemic Count Time Series Model

Shinsuke Koyama[1,2]
[1]Department of Statistical Modeling,
The Institute of Statistical Mathematics, Tokyo, Japan
[2]Department of Statistical Science,
Graduate University for Advanced Studies (SOKENDAI),
Tokyo, Japan

A Derivation of lower bound

From the superposition principle of the Poisson distribution, the conditional probability of secondary infections at (i,t), $Y_{it} = \{y_{it}^{js} : j = 1, \dots, D; s = 1, \dots, t-1\}$, given $N_{1:t-1}$, is obtained as the product of Poisson distributions with the rate $\psi_{it}^{js} = a_{ij} R_{js} \phi_{t-s} n_{js}$,

$$P(Y_{it}|N_{1:t-1}; A, R) = \prod_{j=1}^{D} \prod_{s=1}^{t-1} \frac{\psi_{it}^{js\, y_{it}^{js}}}{y_{it}^{js}!} e^{-\phi_{it}^{js}}, \tag{A.1}$$

and the Poisson distribution (5) for n_{it} is obtained as the marginal distribution of Eq (A.1): Hence, Eq (A.1) can be regarded as a complete data likelihood that contains both observed and latent variables.

In the conventional EM algorithm, which computes the maximum likelihood estimate (MLE) [1], the lower bound is given by the conditional expectation of the complete data likelihood for the entire time series:

$$\begin{aligned} Q(A,R|A^{(k)},R^{(k)}) &= \prod_{i=1}^{D}\prod_{t=1}^{T}\sum_{Y_{it}} P(Y_{it}|N_{1:t}; A^{(k)}, R^{(k)}) \\ &\quad \times \left\{ \sum_{i=1}^{D}\sum_{t=1}^{T} \log P(Y_{it}|N_{1:t-1}; A, R) \right\} \\ &= \sum_{i=1}^{D}\sum_{t=1}^{T}\sum_{j=1}^{D}\sum_{s=1}^{t-1} \Big\{ \langle y_{it}^{js} \rangle^{(k)} \log(a_{ij} R_{js} \phi_{t-s} n_{js}) \\ &\quad - a_{ij} R_{js} \phi_{t-s} n_{js} \Big\}, \end{aligned} \tag{A.2}$$

where we omit the constant terms with respect to the parameters. As the penalty term in the penalized log-likelihood function (9) is convex, the lower bound for the penalized MLE (12) is obtained by adding the penalty term to Eq (A.2).

B Interpretation of the effective reproduction number

To interpret the effective reproduction number $R = \{R_s\}$ defined in model (1), we consider the univariate count time series model ($D = 1$) where the lower bound of (A.2) in the EM algorithm is given by

$$Q(R|R^{(k)}) = \sum_{t=1}^{T} \sum_{s=1}^{t-1} \Big\{ \langle y_t^s \rangle^{(k)} \log(R_s \phi_{t-s} n_s) - R_s \phi_{t-s} n_s \Big\}, \tag{B.1}$$

where y_t^s represents the number of secondary cases at time t that are infected by primary cases at time s ($s < t$). The MLE of the effective reproduction number satisfies the following: $\hat{R} = \arg\max_R Q(R|\hat{R})$,

$$\left. \frac{\partial Q(R|\hat{R})}{\partial R_s} \right|_{R=\hat{R}} = 0 \quad (s = 1, \ldots, T). \tag{B.2}$$

The solution to the above equation is obtained as follows:

$$\hat{R}_s = \frac{1}{n_s} \sum_{t=s+1}^{T} \langle y_t^s \rangle \times \frac{1}{\nu_s}, \tag{B.3}$$

where $\nu_s = \sum_{\tau=1}^{T-s} \phi_\tau$. Eq (B.3) represents the cohort reproduction number, that is, the expected number of secondary infections that an individual who starts showing symptoms at time s will eventually cause. (Here, $1/\nu_s$ is an adjustment for the right truncation with respect to the time interval from infection to reporting and is negligible for $s \ll T$.)

C Equivalent state-space model

To demonstrate the equivalence between the system of equations (14) ($s = 1, \ldots, T$) and the state-space model, we consider a univariate state-space model with a Poisson observation model:

$$p(y_s|x_s) = \text{Poisson}\{\lambda_s(x_s)\} \tag{C.1}$$

and state transition density

$$p(x_s|x_{s-1}) \propto \exp\{-\gamma |x_s - x_{s-1}|^p\}. \tag{C.2}$$

The log joint posterior distribution of state $\{x_1, \ldots, x_T\}$ given a sequence of observations $\{y_1, \ldots, y_T\}$ is then given by

$$\begin{aligned}
&\log p(x_1, \ldots, x_T | y_1, \ldots, y_T) \\
&= \sum_{s=1}^{T} \log p(y_s|x_s) + \sum_{s=2}^{T} \log p(x_s|x_{s-1}) + \text{const} \\
&= \sum_{s=1}^{T} \{y_s \log \lambda_s(x_s) - \lambda_s(x_s)\} - \gamma \sum_{s=2}^{T} |x_s - x_{s-1}|^p + \text{const}, \qquad \text{(C.3)}
\end{aligned}$$

where const. represents constant terms with respect to $\{x_s\}$. The MAP estimate of the states is the value at which the joint posterior distribution (C.3) reaches its maximum value. Therefore, the MAP estimate satisfies the following system of equations:

$$\begin{aligned}
&\frac{\partial}{\partial x_s} \log p(x_1, \ldots, x_T | y_1, \ldots, y_T) \\
&= \frac{y_s - \lambda_s(x_s)}{\lambda_s(x_s)} \frac{\partial \lambda_s(x_s)}{\partial x_s} - \gamma \frac{\partial}{\partial x_s} \{|x_{s+1} - x_s|^p + |x_s - x_{s-1}|^p\} = 0 \qquad \text{(C.4)}
\end{aligned}$$

for $s = 1, \ldots, T$. This can coincide with Eq (14) through the replacement of variables as follows:

$$x_s \to R_{js}, \quad y_s \to \langle y^{js} \rangle^{(k)}, \quad \lambda_s(x_s) \to \nu_s R_{js} n_{js}. \qquad \text{(C.5)}$$

Hence, the solution of the system of equations (14) is equivalent to the MAP estimate of the state-space model (C.1)–(C.2) with replacement (C.5).

D Recursive Bayesian algorithm for state smoothing

The MAP estimate of the state-space model is efficiently computed using a recursive Bayesian algorithm for state smoothing [2, 3]. Here, we describe the algorithm for the state-space model (C.1)–(C.2). The algorithm for computing the solution to Eq (14) is obtained by replacing the variables as per Eq (C.5).

The posterior distribution of x_s given a set of observations up to time s, $y_{1:s} = \{y_1, \ldots, y_s\}$ is obtained using Bayes' theorem as follows:

$$p(x_s|y_{1:s}) = \frac{p(y_s|x_s)p(x_s|y_{1:s-1})}{p(y_s|y_{1:s-1})}, \qquad \text{(D.1)}$$

where

$$p(y_s|y_{1:s-1}) = \int p(y_s|x_s)p(x_s|y_{1:s-1})dx_s. \qquad \text{(D.2)}$$

Here, $p(x_s|y_{1:s-1})$ is obtained from the posterior distribution at time $s-1$, $p(x_{s-1}|y_{1:s-1})$ as

$$p(x_s|y_{1:s-1}) = \int p(x_s|x_{s-1})p(x_{s-1}|y_{1:s-1})dx_{s-1}. \qquad \text{(D.3)}$$

Starting from an initial distribution $p(x_1|y_{1:0}) = p(x_1)$, we iterate Eqs (D.1) and (D.3) to compute $p(x_s|y_{1:s-1})$ and $p(x_s|y_{1:s})$ for $s = 1, 2, \ldots, T$.

Subsequently, we compute the posterior distribution of states x_s ($s = 1, \ldots, T$) given the entire set of observations $y_{1:T} = \{y_1, \ldots, y_T\}$ as

$$p(x_s|y_{1:T}) = p(x_s|y_{1:s}) \int \frac{p(x_{s+1}|y_{1:T})p(x_{s+1}|x_s)}{p(x_{s+1}|y_{1:s})} dx_{s+1}, \quad \text{(D.4)}$$

in the reverse order of $s = T-1, T-2, \ldots, 1$ using $p(x_s|y_{1:s})$ and $p(x_{s+1}|y_{1:s})$ obtained from Eqs (D.1) and (D.3). The MAP estimates of the states and the 95% credible intervals are obtained from the posterior distribution (D.4).

In practice, the integrations in Eqs (D.1), (D.2), and (D.3) are computed numerically. We adopt the numerical algorithm proposed in [4]. The state is discretized into a finite number of segments, and each density function is approximated using a piecewise linear function. The integrations are then approximated as the sum of the values of the density functions evaluated for each segment.

E Free energy of the state-space model

The marginal likelihood function of γ for the state-space model (C.1)–(C.2) is defined as:

$$l(\gamma) = p(y_{1:T}) = \int \cdots \int \left\{ \prod_{s=1}^{T} p(y_s|x_s)p(x_s|x_{s-1}) \right\} dx_1 \cdots dx_T, \quad \text{(E.1)}$$

which is factorized using the product rule as follows:

$$l(\gamma) = \prod_{s=1}^{T} p(y_s|y_{1:s-1}), \quad \text{(E.2)}$$

where $p(y_s|y_{1:s-1})$ is computed using Eq (D.2) with a recursive Bayesian algorithm. The free energy $F(\gamma) = -\log l(\gamma)$ for node j is obtained by replacing the variables according to Eq (C.5).

References

[1] Dempster AP, Laird NM, Rubin DB. Maximum likelihood from Incomplete data via the EM Algorithm. Journal of the Royal Statistical Society Series B (Methodological). 1977;39:1–38.

[2] Durbin J, Koopman S. Time series analysis by state space methods. Oxford University Press; 2001.

[3] Kitagawa G. Introduction to Time Series Modeling. Chapman and Hall/CRC; 2010.

[4] Kitagawa G. Non-Gaussian State-Space Modeling of Nonstationary Time Series. Journal of the American Statistical Association. 1987;82:1032–1041.
