# Supplementary material for: Multivariate epidemic count time series model
Source: PLoS One. 2023 Jun 16;18(6):e0287389. doi: 10.1371/journal.pone.0287389 (PMC10275427; doi:10.1371/journal.pone.0287389)
Supplement: S1 Fig — (PDF) [file pone.0287389.s002.pdf]

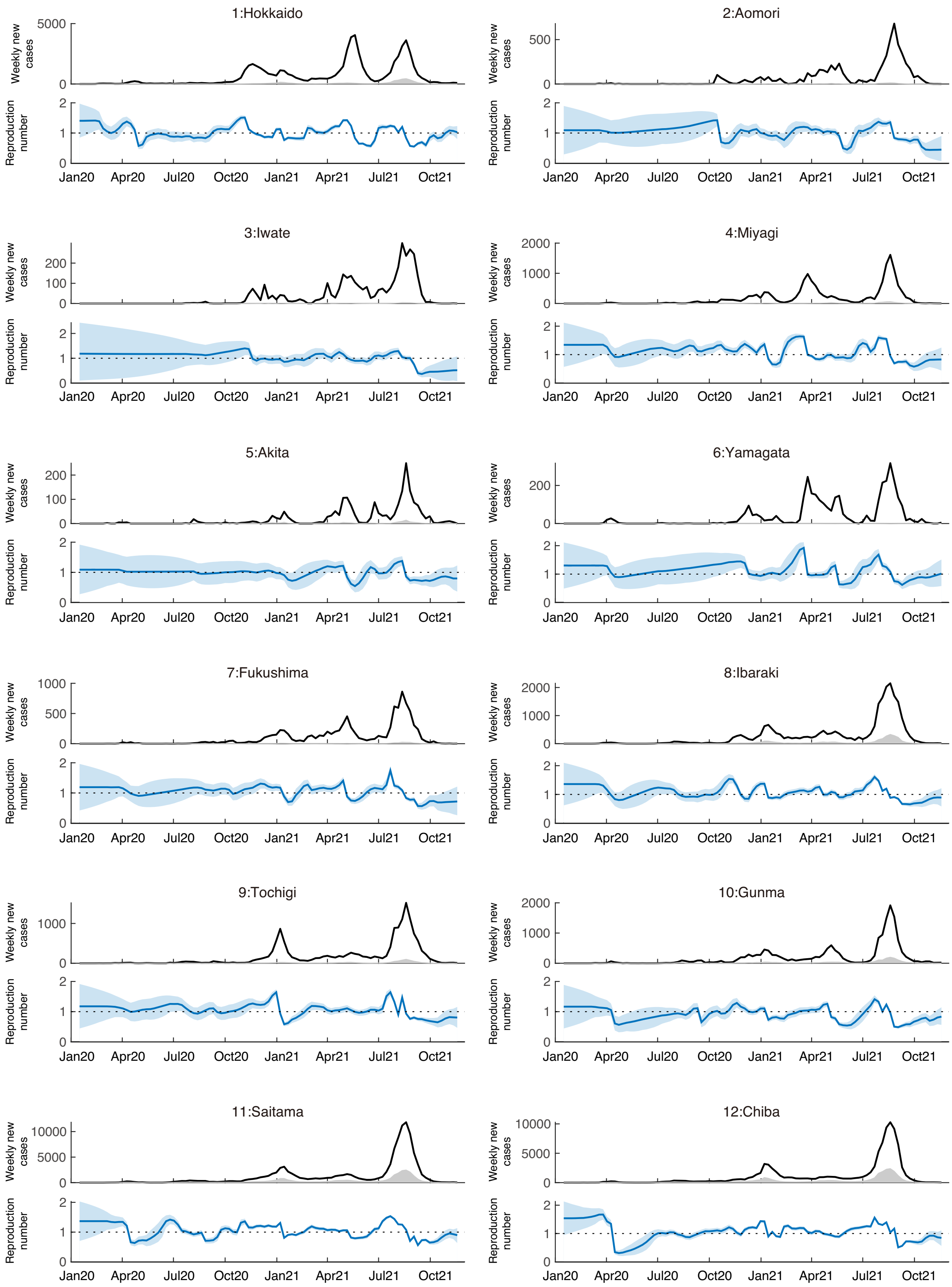

Fig 1. Weekly incidence curve (black line) along with estimated infection counts transmitted from outside (gray area) and estimated time-varying reproduction number (blue line) with 95% credible interval (sky blue) for prefectures 1–12.

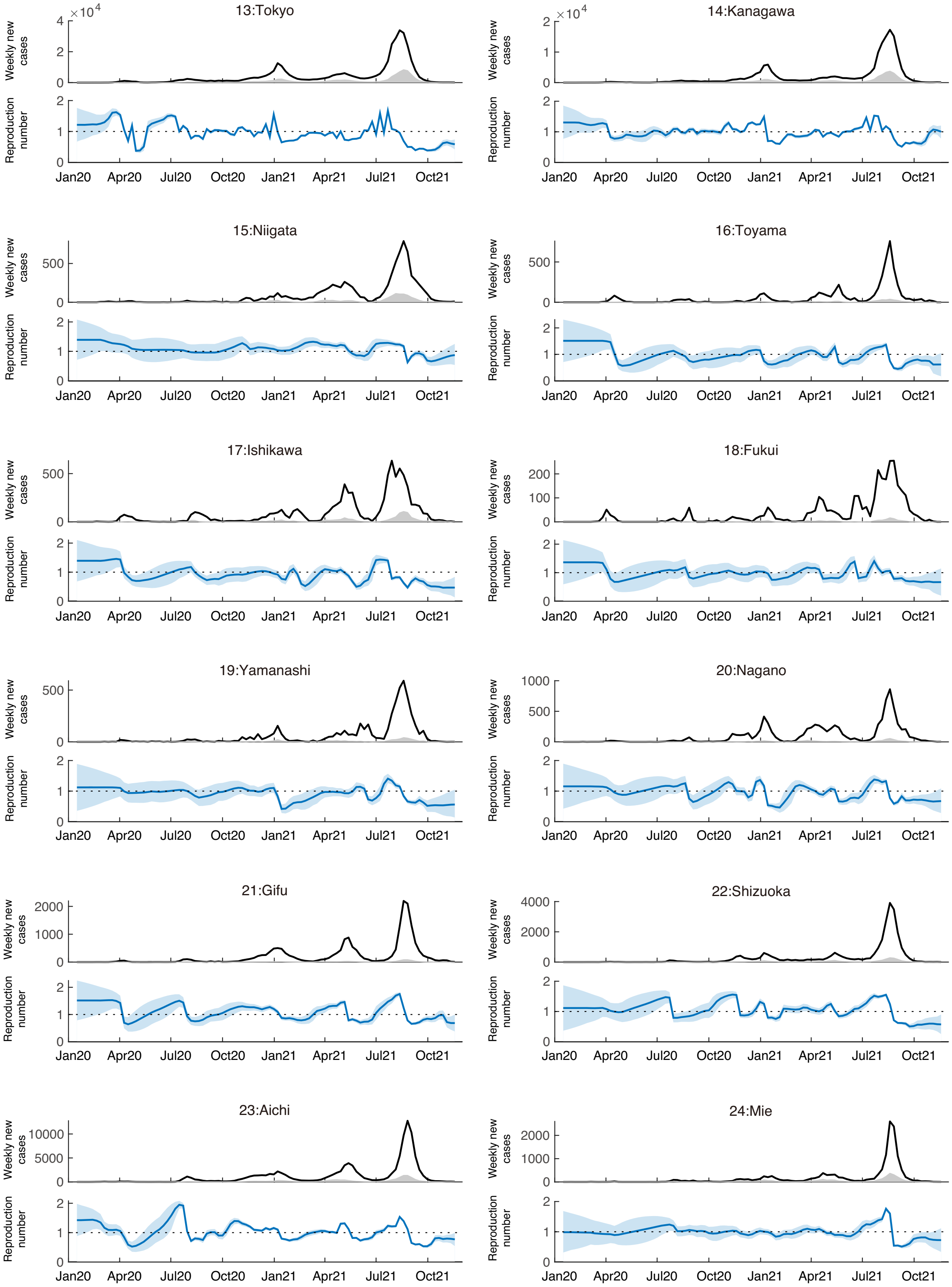

Fig 2. Same as Fig 1 for prefectures 13–24.

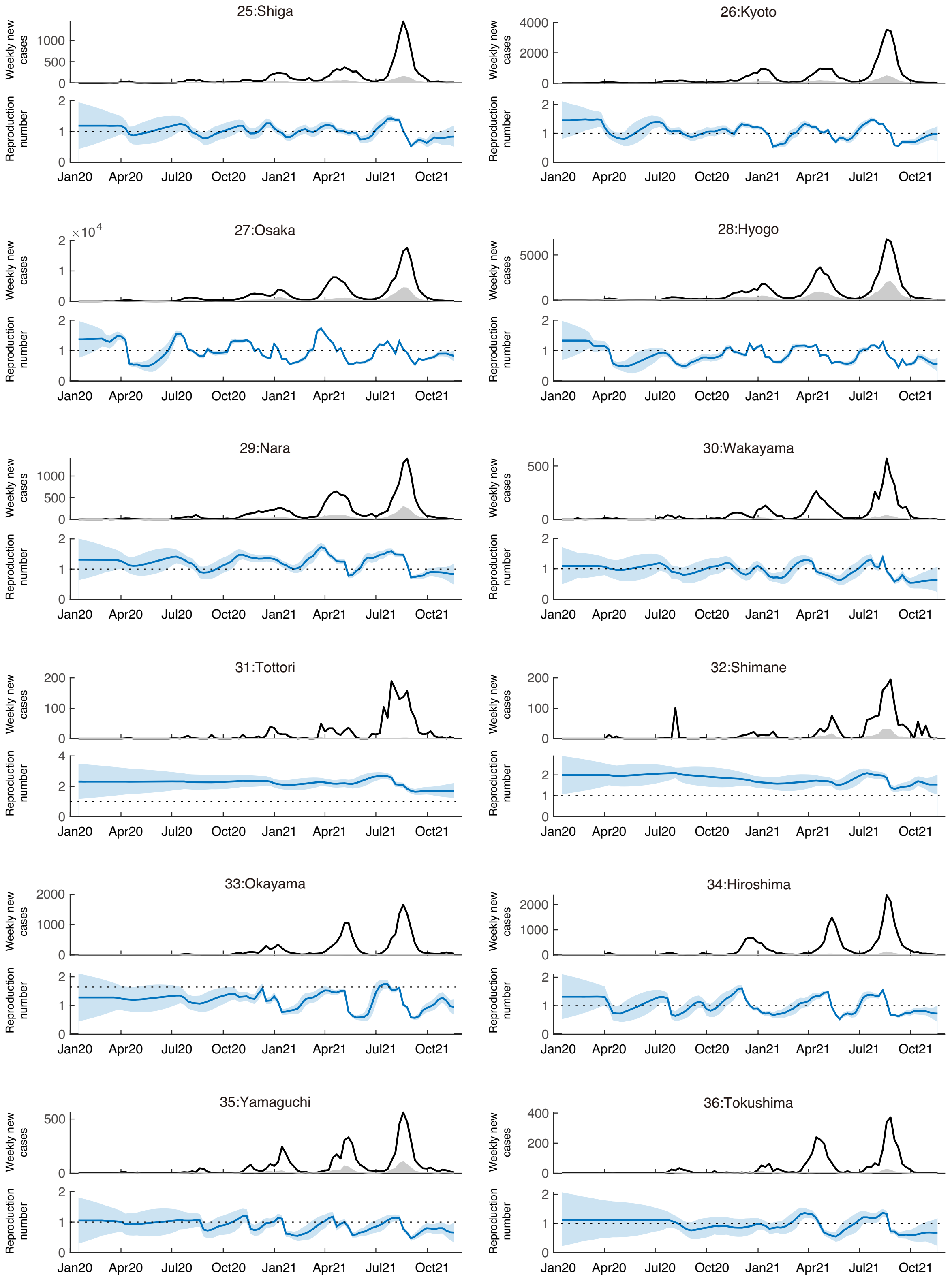

Fig 3. Same as Fig 1 for prefectures 25–36.

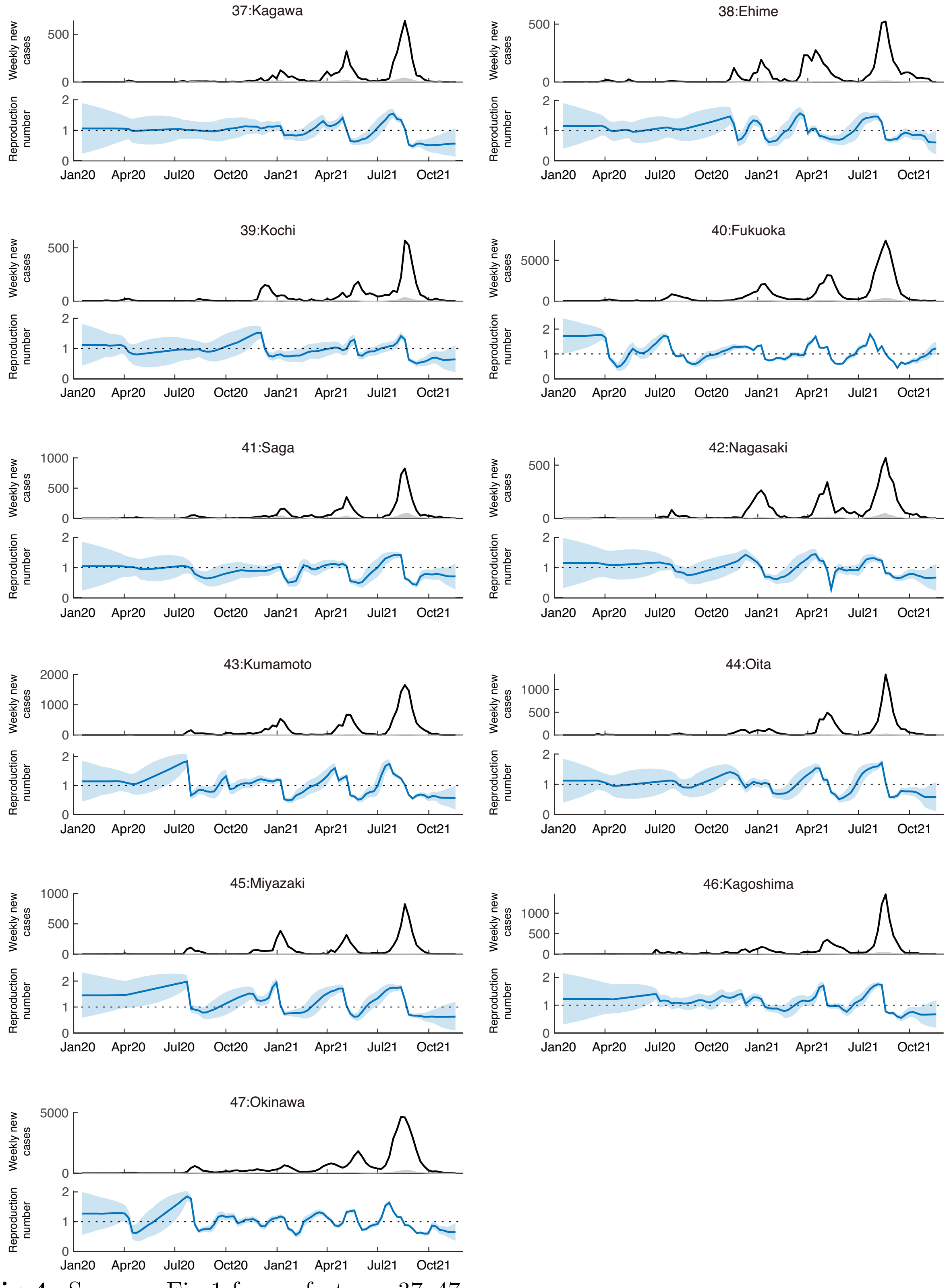

Fig 4. Same as Fig 1 for prefectures 37–47.
